# Comparison of methods for handling missing data on immunohistochemical markers in survival analysis of breast cancer

**DOI:** 10.1038/sj.bjc.6606078

**Published:** 2011-01-25

**Authors:** A M G Ali, S-J Dawson, F M Blows, E Provenzano, I O Ellis, L Baglietto, D Huntsman, C Caldas, P D Pharoah

**Affiliations:** 1 Strangeways Research Laboratory, Department of Public Health and Primary Care, University of Cambridge, Wort's Causeway, Cambridge CB1 8RN, UK; 2 Department of Oncology, University of Cambridge, Cambridge, UK; 3 Cancer Research UK Cambridge Research Institute, Cambridge, UK; 4 Cambridge Breast Unit, Addenbrooke's Hospital, Cambridge University Hospital NHS Foundation Trust and NIHR Cambridge Biomedical Research Centre, Cambridge, UK; 5 Department of Histopathology, Nottingham City Hospital, Nottingham, UK; 6 Cancer Epidemiology Centre, The Cancer Council Victoria, Carlton, Victoria, Australia; 7 Genetic Pathology Evaluation Centre of the Department of Pathology and Prostate Research Centre, Vancouver General Hospital, British Columbia Cancer Agency and University of British Columbia, Vancouver, BC, Canada

**Keywords:** missing data, multiple imputation, complete case analysis, missing covariates, tissue micro-arrays

## Abstract

**Background:**

Tissue micro-arrays (TMAs) are increasingly used to generate data of the molecular phenotype of tumours in clinical epidemiology studies, such as studies of disease prognosis. However, TMA data are particularly prone to missingness. A variety of methods to deal with missing data are available. However, the validity of the various approaches is dependent on the structure of the missing data and there are few empirical studies dealing with missing data from molecular pathology. The purpose of this study was to investigate the results of four commonly used approaches to handling missing data from a large, multi-centre study of the molecular pathological determinants of prognosis in breast cancer.

**Patients and methods:**

We pooled data from over 11 000 cases of invasive breast cancer from five studies that collected information on seven prognostic indicators together with survival time data. We compared the results of a multi-variate Cox regression using four approaches to handling missing data – complete case analysis (CCA), mean substitution (MS) and multiple imputation without inclusion of the outcome (MI−) and multiple imputation with inclusion of the outcome (MI+). We also performed an analysis in which missing data were simulated under different assumptions and the results of the four methods were compared.

**Results:**

Over half the cases had missing data on at least one of the seven variables and 11 percent had missing data on 4 or more. The multi-variate hazard ratio estimates based on multiple imputation models were very similar to those derived after using MS, with similar standard errors. Hazard ratio estimates based on the CCA were only slightly different, but the estimates were less precise as the standard errors were large. However, in data simulated to be missing completely at random (MCAR) or missing at random (MAR), estimates for MI+ were least biased and most accurate, whereas estimates for CCA were most biased and least accurate.

**Conclusion:**

In this study, empirical results from analyses using CCA, MS, MI− and MI+ were similar, although results from CCA were less precise. The results from simulations suggest that in general MI+ is likely to be the best. Given the ease of implementing MI in standard statistical software, the results of MI+ and CCA should be compared in any multi-variate analysis where missing data are a problem.

Missing observations are frequently encountered in all types of epidemiological and clinical studies, no matter how carefully designed or how hard investigators try to prevent the problem (Van der Heijden *et al*, 2006). Missing data are usually classified into three types: missing completely at random (MCAR), missing at random (MAR) and missing not at random (MNAR). Where the probability that an observation is missing is not related to any other subject characteristics, the data are MCAR. This means that whether or not a data point is missing does not depend on observed or unobserved data. Where the probability that an observation is missing depends only on observed information for that subject, the data are MAR. Where the probability that an observation is missing depends on information that is not observed, the data are MNAR (Schafer, 1997; Little and Rubin, 2002; Rubin, 2004).

In clinical epidemiological studies of cancer, tissue micro-arrays (TMAs) are commonly used to generate data on multiple molecular markers in hundreds or thousands of tumours. Tumour characteristics may then be evaluated for association with other clinical or pathological features – disease progression or prognosis is often of particular interest. However, data generated from TMAs are particularly prone to missingness for a variety of technical reasons. Moreover, missingness of TMA data tends to be correlated across variables.

There are three major approaches to handling missing data: deletion-based methods, in which observations with missing data are excluded from the analysis, simple replacement methods, in which the missing data are replaced by plausible values based on available data for that variable, and model-based or conditional methods, where the missing data are replaced by values estimated from models that utilise the available data for all the variables in the dataset. The most commonly used method for dealing with missing data is complete case analysis (CCA), a deletion-based method. CCA may be preferred under the situation in which the sample size is large, the proportion of missing data is small and the missing data mechanism is approximately MCAR (Kim and Curry, 1977). However, even when data are MCAR, loss of data will result in loss of precision (larger standard errors), particularly with multivariate data analysis. Where data are MAR or MNAR, CCA can be biased (Little, 1992; Greenland and Finkle, 1995; Schafer and Graham, 2002).

An alternative approach is to use methods that fill-in, or impute, the missing data. Among the simple replacement methods, mean substitution (MS), which is also known as overall mean imputation, is commonly used. It is a simple, unconditional method that does not depend on observed data for other variables in the imputation. However, MS may also result in biased estimated where data are not MCAR, but the estimates are more precise than with CCA (Donders *et al*, 2006). A somewhat more sophisticated approach is to fill in missing values for each subject with values predicted from the rest of the data (model-based methods or conditional imputation). When missing data are not MCAR, single conditional imputation tends to result in less biased parameter estimates than CCA or MS, but the standard errors will be underestimated if the data are treated as a dataset with no missing values (Greenland and Finkle, 1995; Van der Heijden *et al*, 2006). Formulae for correcting the standard errors for single conditional imputation can be complex and multiple imputation (MI) has been proposed as an alternative. In MI, multiple copies of each dataset are generated, each with missing values replaced by values randomly generated under a specified model that utilises the rest of the data. Each dataset is analysed as if it were complete and the results from each are then combined in a manner that takes account of imputation variability to obtain the correct standard errors. MI is now available in standard statistical software and is relatively straightforward to implement. MI, like other imputation methods, produces parameter estimates that are more precise than CCA and has also been shown in many studies to generate unbiased results under a variety of scenarios for missing data (Greenland and Finkle, 1995; Van der Heijden *et al*, 2006; Ambler *et al*, 2007). Nevertheless, MI may not be superior under some circumstances (van Buuren *et al*, 1999). It is thus apparent that no method of dealing with missing data is appropriate under all conditions, and the best approach will depend on the degree of data missingness and the structure of the underlying missing data – correlations of missing covariate and outcome data.

Different approaches have often been compared using simulated data. However, there are few published studies that have compared methods using large, empirical datasets. Given that the validity of different approaches depends on the structure of missing data in a given dataset, it is important to evaluate different methods using a variety of empirical data. Ambler *et al* (2007) compared different methods using a large dataset investigating outcome after cardiac surgery. Correlations between covariates in this dataset were weak. The results suggested that CCA produced unreliable risk estimates, whereas the results of MI were more accurate. However, they suggested further research based on data with stronger correlations between variables and speculated that MI would also perform best under these circumstances.

Dawson *et al* (2010) recently published an evaluation of the role of oestrogen receptor (ER), progesterone receptor (PR), human epidermal growth factor receptor-2 (HER2) and B-cell lymphoma 2 (BCL2) expressions in the prognosis of breast cancer using data generated from TMAs with tumours from more than 11 000 breast cancer cases from five studies. The main multi-variate analysis of these data was restricted to the 5443 subjects with complete data – a CCA. The purpose of the analyses we report in this paper was to evaluate the structure of the missing data in this dataset and to compare the results of analyses of this dataset using different imputation approaches to deal with the missing data.

## Patients and methods

### Study population

We used data from a study on prognosis of breast cancer for which methods and results have been described (Dawson *et al*, 2010). In brief, a total of 11 212 early-stage breast cancer cases were participants in five different studies; The Study of Epidemiology and Risk Factors in Cancer Heredity (SEARCH), the Nottingham Breast Cancer Series (NBCS), University of British Columbia Breast Cancer Series (UBCBCS), British Columbia Cancer Agency Case Series (BCCA) and Melbourne Collaborative Cohort Study (MCCS). All studies were approved by the relevant institutional review boards or ethics committees. The criteria for inclusion were the availability of tumour tissue, pathological data (tumour size, tumour grade, lymph node status) and individual clinical outcome data (breast cancer-specific death). Early-stage breast cancer was defined as stage I to stage III as per Greene *et al* (2002).

Breast cancer-specific mortality was the end point of interest, and was defined as a death where breast cancer was given as the underlying cause on the death certificate. Seven variables were included in the prognostic model: nodal status, tumour size, histopathological grade, ER status, PR status, HER2 status and BCL2 status.

### Statistical analysis

We used the two approaches suggested by Little and Rubin (2002) to assess the randomness of the missing data in our dataset. Firstly, prognosis was compared in cases with and without missing data for each variable. If the data were MCAR, there would be no difference between the groups. We also assessed the correlation of data missingness for each pair of variables, which is expected to be uncorrelated for data MCAR. No guidelines exist for identifying the level of correlation needed to indicate that the missing data are not MCAR. Statistical significance tests of the correlations provide a conservative estimate of the degree of randomness. Significant correlations in missingness between some pairs of variables suggest that the data are MAR or MNAR.

A dataset with MS of missing values was generated by simply replacing missing values with the mean of the available data for that variable. Multiply-imputed datasets were generated using the *ice* command in Stata (Stata Corporation, College Station, TX, USA), which imputes missing values by using switching regression, an iterative multivariable regression technique. Missing data theorists have claimed that good inferences can be made with the number of imputed datasets (*m*) as few as 3–5 (Rubin, 2004). They have argued that the relative efficiency of estimation is very high under these circumstances compared with an infinite number of imputations. However, others have suggested that (*m*) should approximate the percentage of subjects with some missing data (Bodner, 2008). We used *m*=50 because about half of the cases in the dataset of this study have incomplete data. All seven prognostic variables were included in the data imputation model. Dichotomous covariates (ER, PR, HER2, BCL2 and node status) were imputed using logistic regression and polychotomous covariates (grade and tumour size) were imputed using polytomous logistic regression. It was suggested that MI models that ignore the outcome of interest can bias parameter estimates towards the null (Moons *et al*, 2006; White and Royston, 2009) and so we also imputed the missing data, including the outcome of interest in the imputation model.

Cox regression analysis stratified by the study was performed to determine the effect of each prognostic factor and marker on breast cancer-specific survival after diagnosis. Hazard ratio estimates were calculated over the 15-year follow-up period using the Cox model that included all seven prognostic variables. Tests of the proportional hazards assumption based on Schoenfeld residuals were not significant for tumour size (*P*=0.08), HER2 status (*P*=0.62) or BCL2 status (*P*=0.2), but were significant for nodal status (*P*=0.01), grade (*P*<0.0001), ER status (*P*<0.0001) and PR status (*P*<0.0001). These four variables were therefore treated as time-dependent variables in an extended Cox model in subsequent analyses. Cox regression was performed on four datasets: the dataset with complete data, the dataset in which missing data were replaced by the mean (MS) and the multiply-imputed datasets – those imputed with and without including survival time in the imputation model (MI+ and MI−). The analysis of the multiply-imputed datasets was carried out using the *micombine* command in Stata.

### Simulations

To provide additional evidence for the results, we simulated 100 datasets with data MCAR and 100 datasets with data MAR using the subset of cases with no missing data (*n*=5443), and to simulate sampling variation, as well as variation in missing data, we generated each dataset by bootstrap sampling with replacement from the complete dataset, and then simulated the missing data on the bootstrap datasets. We generated the data MCAR by deleting data for each variable randomly in proportion to the missingness of the data in the full dataset. The proportion of cases with complete data in each simulated dataset was lower than that in the full dataset – 27 percent (1447 of 5443) compared with 49 percent (5443 of 11 212). This is as expected where there is no correlation between missing data. We generated data MAR with the same pattern of missingness as in the original dataset, so that the correlations between missingness in the original dataset were preserved. In these datasets, the proportion of complete cases (2593 of 5443) was the same as in the original data. Each of the simulated datasets was analysed using the four approaches described above. Cox regression analysis including time-dependent variables as described above is relatively slow – the multivariate analysis of 50 multiply-imputed datasets taking about 12 h. To reduce computation time for the analysis of 200 simulated datasets, we restricted the analysis to the first 2 years of follow-up as the proportional hazards assumption is reasonably robust over such a short time span. We compared the estimates from the simulated datasets with the estimates from the analysis of the complete dataset.

All statistical analyses were run using Stata SE10.

## Results

Baseline clinical and pathology data from the study subjects are summarised in Table 1. All seven variables used in the model had missing values. Of the 78 484 possible data points, 13 357 were missing (6 percent) in 5769 patients (51 percent). Table 2 shows that the least data were missing for ER status (6 per cent) and the most data were missing for BCL2 status (30 percent). Supplementary Table 1 shows the number of subjects according to the number of missing data points. Almost half the subjects (5443) had a complete set of data for all seven variables, and 47 subjects (0.4 percent) had data on only one variable.

There was a significant difference in survival of patients with and without missing data for all four IHC markers (*P*=0.005, 0.04, 0.001 and 0.04 for ER, PR, HER2 and BCL2 status, respectively) (Supplementary Figure 1). Those patients with missing data had a better prognosis, so exclusion of the patients with missing values would lead to an underestimate of the true survival of the cohort. A likely explanation is that tumour cores are more likely to be missing from a TMA if the tumour is small and difficult to sample. Survival in patients missing tumour grade was better than those with available data (*P*<0.001). For nodal status and size of the tumour, the reverse was true, that is, survival in patients missing nodal status (*P*<0.0001) and size of the tumour (*P*=0.075) was worse than those with available data (Supplementary Figure 2).

The overall number of subjects with complete data would be expected to be 27 percent of the total if the missing data were not correlated. That 49 percent of cases had complete data suggest that missingness is correlated between variables and thus the data are either MAR or MNAR. The correlation between data missingness for all prognostic factors shows clear correlations between missingness for most pairs (Table 3). In particular, the strongest correlations were between the immunohistochemical markers, as might be expected for data generated from TMAs. Taken together, these findings strongly suggest that the data are not MCAR.

The model coefficients (natural logarithm of hazard ratio) and standard errors from the multi-variate Cox regression based on the four approaches to handling missing data are shown in Table 4. There was little difference in the coefficient estimates for the four methods, but the standard errors for the CCA were substantially larger, as would be expected from the smaller sample size. Note that the underlying ‘true’ estimates for comparison are not known.

### Results of the simulations

In contrast, the results from the analyses of the datasets with simulated missing data were somewhat different. Here the estimates from the analysis of the 5443 subjects with complete data are regarded as the underlying ‘true’ estimates for comparison with the results from CCA, MS, MI− and MI+ analyses of datasets with missing data simulated as MCAR and MAR.

**Figure 1:**
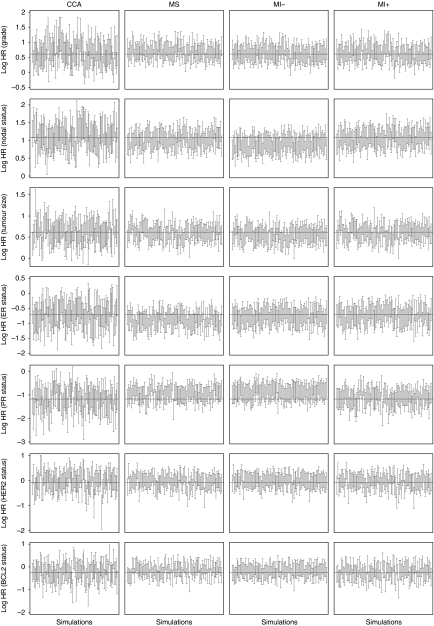
Confidence limits for multivariate log hazard ratio estimates for each prognostic variable using four approaches of handling missing data in 100 datasets with data-simulated MAR. CCA=complete case analysis; MS=mean substitution; MI=multiple imputation without the outcome and MI+=multiple imputation with the outcome. The horizontal lines represent the true estimates.

Supplementary Figure 3 shows the model coefficients for the 200 datasets with missing data simulated as MCAR and MAR under the four analytic approaches. The difference between these values and the true value provides an indication of the accuracy and bias of the analytic approach (Engels and Diehr, 2003). The mean of the absolute difference provides a measure of accuracy – how close the estimated values are to the true value. The mean of the difference provides a measure of whether the estimates tend to be systematically over- or underestimated, or biased. These results (Table 5) show that the values for CCA were more likely to be extreme than for the other three methods, but the distribution of these values tended to be unbiased. The values for the other three methods tended to be less extreme, but for some variables there was evidence for bias. In particular, for the data-simulated MAR, the estimates from MS for grade tended to be overestimated, whereas those for ER status and PR status tended to be underestimated. Similarly for MI−, the HR for nodal status tended to be underestimated, whereas HR for PR status tended to be overestimated. The most consistent results were obtained using MI+ with little evidence for bias. Figure 1 shows the 95 percent confidence intervals (CIs) for each log HR estimate for the 100 data MAR simulations. The CIs for the results of the data simulated as MCAR are shown in Supplementary Figure 4. This shows that the CIs for CCA tend to be wider than for the other three methods. For the data MCAR, the CIs for the CCA, MS, MI− and MI+ excluded the true value 31, 64, 47 and 26 times, respectively, out of 700 estimates (7 variables and 100 simulated datasets). For the data MAR, the CIs excluded the true value 73, 96, 67 and 48 times, respectively, for CCA, MS, MI− and MI+.

## Discussion

The occurrence of missing data is a pervasive problem in the analysis of epidemiological data. Several possible techniques can be used to deal with the problem, but the appropriateness of the methods may depend on the nature of the missing data. We have evaluated four commonly used methods in the analysis of molecular pathology data generated using TMAs in the study of cancer. Such data are particularly prone to correlated missingness.

As expected, the missing data for the seven variables of interest in our dataset were correlated with each other and with the outcome of interest, suggesting that the data are MAR or MNAR. Note that it is not possible to determine which of these possibilities is correct using the observed data. Under these circumstances, we might expect MI+ with the model used to impute missing data, including the outcome of interest to provide the least biased parameter estimates, with a potential for bias for estimates based on CCA, MS and MI−. However, we found little difference in the model coefficient estimates for the analyses of the full dataset of 11 212 subjects using CCA and both methods of missing data imputation. This suggests that all four methods generate estimates that are reasonably robust with large sample sizes, even where a substantial proportion of the data are missing. The coefficient standard errors were greater for CCA than for either of the imputation methods, as would be expected from the considerable loss of sample size that occurs for CCA.

In contrast, the results of the analysis of the datasets with missing data simulated as MCAR and MAR varied according to the approach used. There was no systematic trend for the results of CCA to either over- or underestimate the true value, that is, there was no bias, but the CIs were wider than with other approaches. All seven variables used are established as prognostic factors, and despite the loss of precision, all the associations were significant at the 5 percent level. However, with a smaller sample size and/or smaller effects, some associations might not be detected by CCA because of the loss of statistical power. Furthermore, as the number of variables increases, even a small proportion of missing data for each variable can lead to severely depleted data for CCA and substantial loss of power. MS and MI− gave similar results. Confidence limits were small and the point estimates tended to be closer to the true estimate than with CCA. On the other hand, MS and MI− systematically under- or overestimated the effects for some variables.

As shown by others (Rubin and Schenker, 1991; Vach, 1991; Greenland and Finkle, 1995; Rubin, 1996, 2004; Schafer, 1999; Little and Rubin, 2002), our results show that MI without the outcome variable in the imputation model results in bias towards the null for the regression coefficients. This reflects the fact that missingness of a predictor is commonly related to both other predictors (covariates) and, directly or indirectly, to the outcome. Consequently, the estimated conditional distribution of the covariate from which random values are drawn (multiple times) to replace the missing covariate values will be biased unless the outcome is included in the imputation mode (Moons *et al*, 2006).

It has been suggested that where more than 25 percent of cases have missing data, the loss of precision from CCA is sufficient to warrant an alternative approach to analysis (Marshall *et al*, 2010). However, it is not possible to rank definitively one method over the other as the choice depends on the relative importance given to accuracy and bias. There are many factors that may affect the performance of the different approaches. These include the sample size, number of variables of interest, the proportion of missing data for each variable, the correlation between variables, the correlation between data missingness for the variables and the underlying association between each variable and the outcome of interest. As pointed out by Horton *et al* (2010), all missing data methods rely on inherently untestable assumptions, and we cannot draw general conclusions on the most appropriate method of handling missing data in multivariable prediction research under all circumstances. Guidelines for reporting analyses potentially affected by missing data have recently been published and our results support their main conclusions (Sterne *et al*, 2009). In particular, a comparison of the results of different approaches to handling missing data should be considered.

In conclusion, the results from our empirical analyses using CCA, MS, MI− and MI+ were similar, although results from CCA were less precise. The results from simulations suggest that, in general, MI+ gave consistently more accurate estimates with the least bias. Given the ease of implementing MI in standard statistical software, the results of MI+ and CCA should be compared in any multi-variate analysis where missing data are a problem.

## Figures and Tables

**Table 1:** Baseline characteristics of breast cancer datasets

**Variable**	**Number (percent)**
Mean age	55 (N/A)
Mean follow-up	8.4 (N/A)
Number of breast deaths	2677 (24)
5-year survival	8002 (71)
	
*Grade*	
1	1437 (13)
2	4155 (36)
3	4546 (41)
Missing	1074 (10)
	
*Nodal status*	
Negative	5478 (49)
Positive	4060 (36)
Missing	1674 (15)
	
*Tumour size (cm)*	
<2	4545 (41)
2–4.9	4664 (41)
5+	697 (6)
Missing	1306 (12)
	
*ER status*	
Negative	3037 (27)
Positive	7458 (67)
Missing	717 (6)
	
*PR status*	
Negative	3963 (35)
Positive	5030 (45)
Missing	2219 (20)
	
*HER2 status*	
Negative	7068 (63)
Positive	1104 (10)
Missing	3040 (27)
	
*BCL2 status*	
Negative	2185 (19)
Positive	5700 (51)
Missing	3327 (30)

Abbreviations: BCL2=B-cell lymphoma 2; ER=oestrogen receptor; HER2=human epidermal growth factor receptor-2; N/A=not applicable; PR=progesterone receptor.

**Table 2:** Number of missing values for prognostic covariates

**Variable**	**Observed**	**Missing (% )**
Grade	10 138	1074 (10)
Nodal status	9538	1674 (15)
Tumour size	9906	1306 (12)
ER status	10 495	717 (6)
PR status	8993	2219 (20)
HER2 status	8172	3040 (27)
BCL2 status	7885	3327 (30)

Abbreviations: BCL2=B-cell lymphoma 2; ER=oestrogen receptor; HER2=human epidermal growth factor receptor-2; PR=progesterone receptor.

**Table 3:** Correlation of missingness in breast cancer prognostic factors

	**Grade**	**Nodal status**	**Size group**	**ER status**	**PR status**	**HER2 status**
Grade	1.00					
Nodal status	0.09	1.00				
Size group	0.10	0.73	1.00			
ER status	0.01	−0.03	(0.01)	1.00		
PR status	(−0.01)	0.08	0.07	0.41	1.00	
HER2 status	(0.01)	0.28	0.23	0.36	0.57	1.00
BCL2 status	−0.04	0.07	0.05	0.30	0.58	0.57

Abbreviations: BCL2=B-cell lymphoma 2; ER=oestrogen receptor; HER2=human epidermal growth factor receptor-2; PR=progesterone receptor.

Coefficients within parentheses are not statistically significant (*P*>0.05).

**Table 4:** Comparison of coefficients (log hazard ratio) and standard errors (s.e.) from analyses based on four methods for handling missing data

	**Complete case analysis**		**Mean substitution**		**MI−**		**MI+**	
**Variable**	**Coefficient**	**s.e.**	**Coefficient**	**s.e.**	**Coefficient**	**s.e.**	**Coefficient**	**s.e.**
Grade	1.05	0.14	1.05	0.10	0.95	0.10	0.97	0.10
Nodal status	1.17	0.13	0.97	0.09	0.97	0.10	1.10	0.10
Tumour size	0.41	0.05	0.45	0.03	0.40	0.04	0.43	0.04
ER status	−0.89	0.15	−0.93	0.11	−0.83	0.11	−0.80	0.11
PR status	−1.13	0.16	−1.00	0.12	−0.94	0.12	−1.00	0.13
HER2 status	0.27	0.07	0.27	0.05	0.23	0.05	0.26	0.05
BCL2 status	−0.26	0.07	−0.20	0.06	−0.17	0.06	−0.20	0.06
Time effect								
Nodal status	−0.22	0.08	−0.13	0.06	−0.19	0.06	−0.19	0.06
Grade	−0.40	0.08	−0.37	0.06	−0.34	0.06	−0.33	0.06
ER status	0.71	0.10	0.68	0.07	0.63	0.08	0.63	0.08
PR status	0.55	0.10	0.49	0.08	0.46	0.08	0.47	0.08

Abbreviations: BCL2=B-cell lymphoma 2; ER=oestrogen receptor; HER2=human epidermal growth factor receptor-2; MI−=multiple imputation without the outcome; MI+=multiple imputation with the outcome; PR=progesterone receptor.

**Table 5:** MD and MAD for each imputation method, averaged over 100 simulations

	**MCAR**				**MAR**			
	**CCA**	**MS**	**MI−**	**MI+**	**CCA**	**MS**	**MI−**	**MI+**
*MD*								
Grade	−0.06	−0.06	−**0.01**	−0.03	−0.04	−0.08	−**0.02**	−0.03
Nodal status	**0.02**	0.03	0.18	**0.02**	−**0.02**	0.07	0.20	**0.02**
Tumour size	−0.01	−0.02	0.04	**0.00**	**0.00**	0.02	0.06	0.01
ER status	0.02	0.14	0.05	**0.01**	**0.01**	0.17	0.06	0.02
PR status	−0.09	−0.20	−0.27	−**0.08**	**0.03**	−0.22	−0.30	−0.05
HER2 status	−**0.01**	−0.04	−0.03	0.04	−**0.01**	−**0.01**	−0.05	−**0.01**
BCL2 status	**0.02**	0.04	−**0.02**	0.03	**0.00**	−0.09	−0.06	−0.01
								
*MAD*								
Grade	0.27	0.13	**0.12**	0.14	0.22	**0.11**	**0.11**	0.14
Nodal status	0.20	0.12	0.18	**0.11**	0.20	0.20	0.20	**0.12**
Tumour size	0.16	0.09	**0.08**	0.09	0.15	**0.08**	0.11	0.11
ER status	0.25	0.17	**0.12**	0.14	0.23	0.20	**0.14**	0.15
PR status	0.28	0.24	0.28	**0.18**	0.29	0.26	0.30	**0.18**
HER2 status	0.28	0.14	**0.10**	0.14	0.22	0.15	**0.12**	0.16
BCL2 status	0.24	0.14	**0.11**	0.15	0.23	0.19	**0.14**	0.18

Abbreviations: BCL2=B-cell lymphoma 2; CCA=complete case analysis; ER=oestrogen receptor; HER2=human epidermal growth factor receptor-2; MAD=mean absolute difference; MAR=missing at random; MCAR=missing completely at random; MD=mean deviation; MI−=multiple imputation without the outcome; MI+=multiple imputation with the outcome; MS=mean substitution; PR=progesterone receptor.

Numbers in bold indicate method with best result for that variable. Underlined numbers indicate method with worst result for that variable.
