# Supplementary figures and images for: Comparison of methods for handling missing data on immunohistochemical markers in survival analysis of breast cancer
Source: Br J Cancer. 2011 Jan 25;104(4):693–9. doi: 10.1038/sj.bjc.6606078 (PMC3049587; doi:10.1038/sj.bjc.6606078)

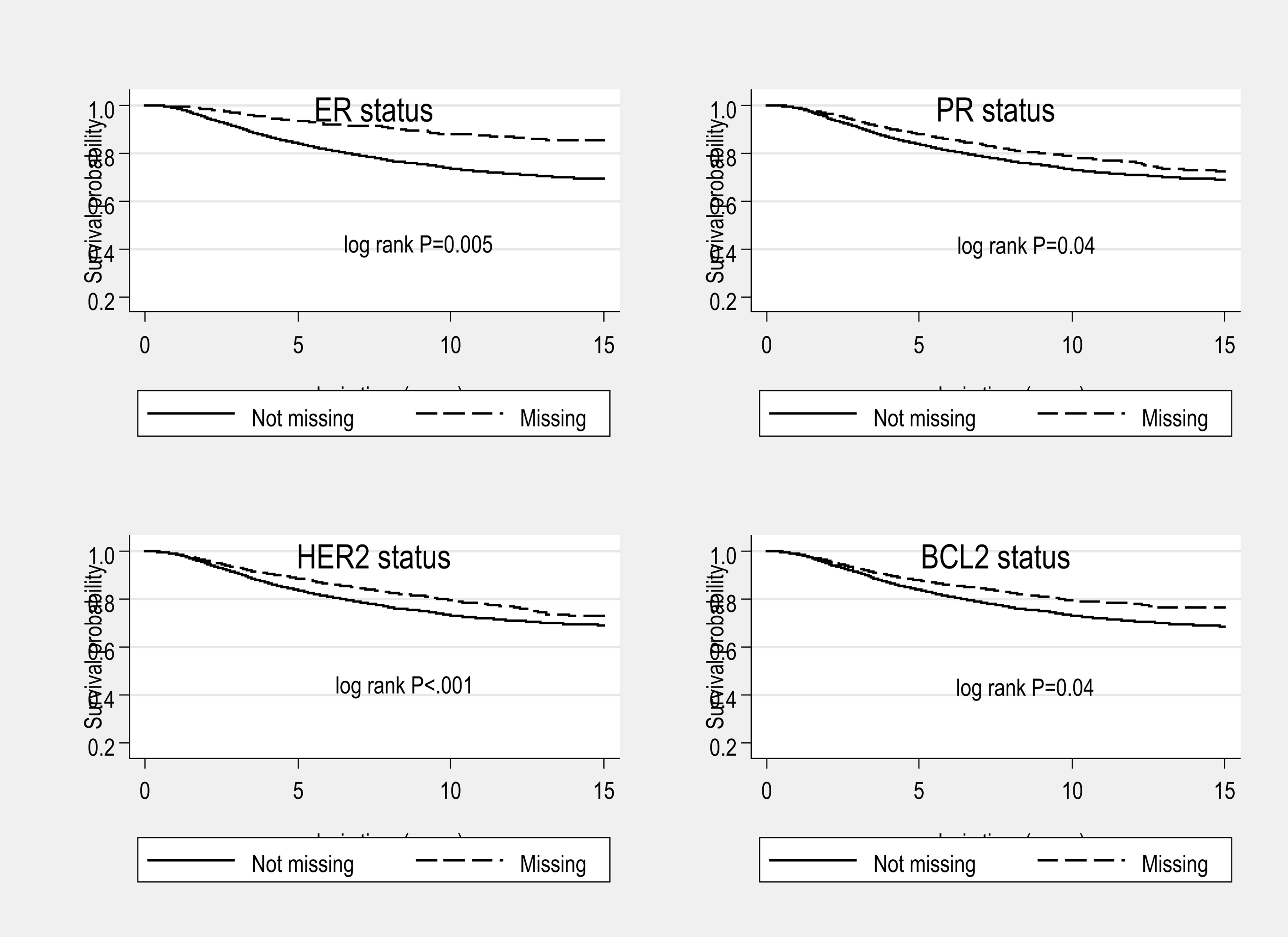

Supplement: Supplementary Figure 1 [file 6606078x1.tif]

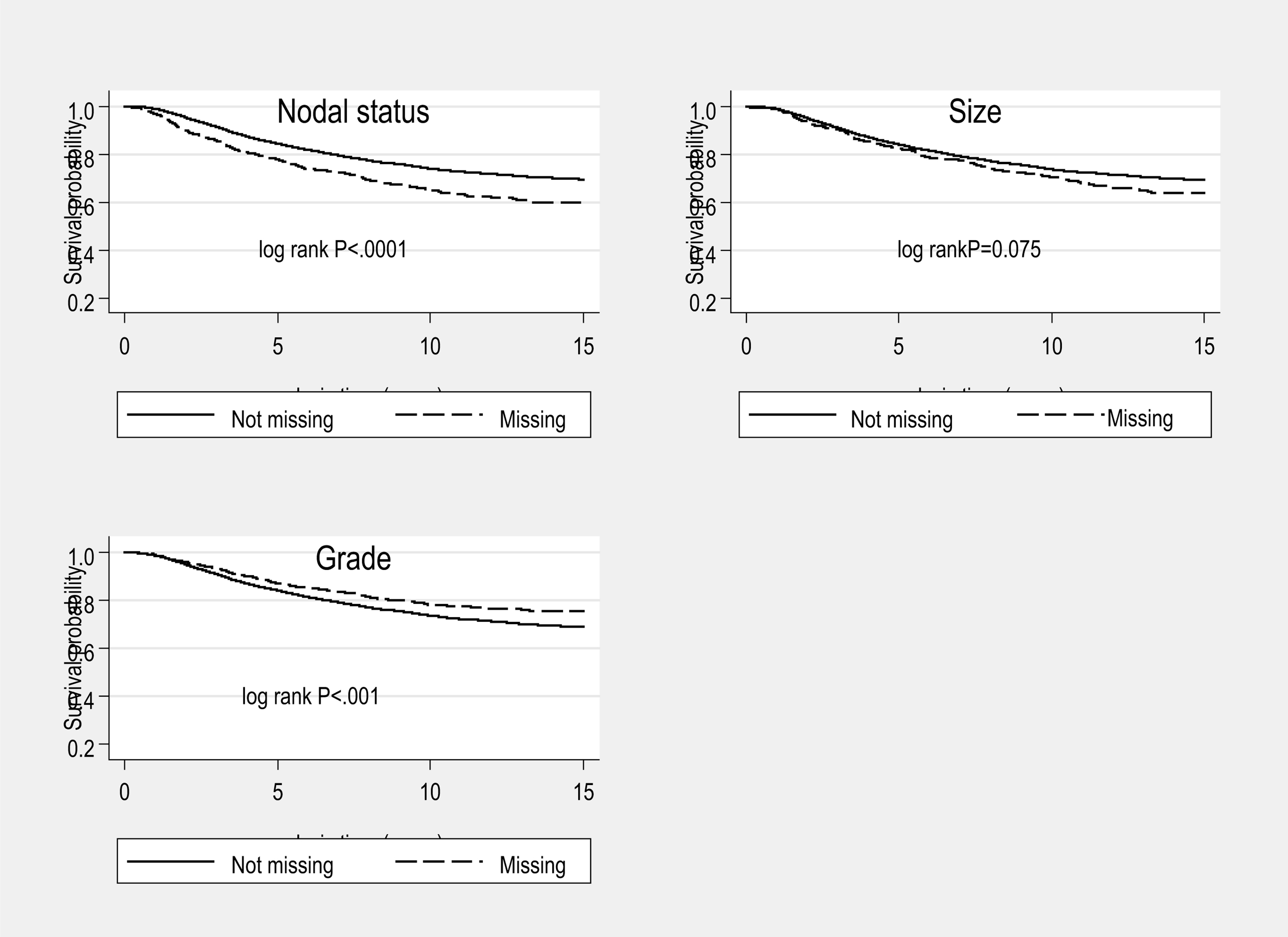

Supplement: Supplementary Figure 2 [file 6606078x2.tif]
