# Supplementary material for: Comparison of methods for handling missing data on immunohistochemical markers in survival analysis of breast cancer
Source: Br J Cancer. 2011 Jan 25;104(4):693–9. doi: 10.1038/sj.bjc.6606078 (PMC3049587; doi:10.1038/sj.bjc.6606078)
Supplement: Supplementary Table 1 [file 6606078x5.doc]

**Supplementary table 1 Number** of subjects by number of missing data points

| **Missing** | **Frequency** | **Percent** |
| --- | --- | --- |
| 0 | 5,443 | 49 |
| 1 | 2,211 | 20 |
| 2 | 1,181 | 11 |
| 3 | 1,204 | 11 |
| 4 | 740 | 6.6 |
| 5 | 386 | 3.4 |
| 6 | 47 | 0.4 |
| Total | 11,212 | 100 |
